# COVID-19: Direct and Indirect Mechanisms of Statins

**DOI:** 10.3390/ijms22084177

**Published:** 2021-04-17

**Authors:** Agnieszka Pawlos, Mateusz Niedzielski, Paulina Gorzelak-Pabiś, Marlena Broncel, Ewelina Woźniak

**Affiliations:** Laboratory of Tissue Immunopharmacology, Department of Internal Diseases and Clinical Pharmacology, Medical University of Lodz, Kniaziewicza 1/5, 91-347 Lodz, Poland; mateusz.niedzielski@stud.umed.lodz.pl (M.N.); paulina.gorzelak-pabis@umed.lodz.pl (P.G.-P.); marlena.broncel@umed.lodz.pl (M.B.); ewelina.wozniak@umed.lodz.pl (E.W.)

**Keywords:** statins, COVID-19, SARS-CoV-2, macrophages activation syndrome (MAS), main protease (Mpro), RNA-dependent RNA polymerase (RdRp), TLR 4, IL-6, ACE2, thrombosis

## Abstract

The virus responsible for the current COVID-19 pandemic is severe acute respiratory syndrome coronavirus 2 (SARS-CoV-2): a new virus with high infectivity and moderate mortality. The major clinical manifestation of COVID-19 is interstitial pneumonia, which may progress to acute respiratory distress syndrome (ARDS). However, the disease causes a potent systemic hyperin-flammatory response, i.e., a cytokine storm or macrophage activation syndrome (MAS), which is associated with thrombotic complications. The complexity of the disease requires appropriate intensive treatment. One of promising treatment is statin administration, these being 3-hydroxy-3-methylglutaryl-CoA reductase inhibitors that exert pleiotropic anti-inflammatory effects. Recent studies indicate that statin therapy is associated with decreased mortality in COVID-19, which may be caused by direct and indirect mechanisms. According to literature data, statins can limit SARS-CoV-2 cell entry and replication by inhibiting the main protease (Mpro) and RNA-dependent RNA polymerase (RdRp). The cytokine storm can be ameliorated by lowering serum IL-6 levels; this can be achieved by inhibiting Toll-like receptor 4 (TLR4) and modulating macrophage activity. Statins can also reduce the complications of COVID-19, such as thrombosis and pulmonary fibrosis, by reducing serum PAI-1 levels, attenuating TGF-β and VEGF in lung tissue, and improving endothelial function. Despite these benefits, statin therapy may have side effects that should be considered, such as elevated creatinine kinase (CK), liver enzyme and serum glucose levels, which are already elevated in severe COVID-19 infection. The present study analyzes the latest findings regarding the benefits and limitations of statin therapy in patients with COVID-19.

## 1. Introduction

SARS-CoV-2 is a novel coronavirus, and one responsible for the COVID-19 pandemic. The first official reports from Wuhan City, China about numerous cases of acute, severe respiratory syndrome appeared in December 2019, and the coronavirus itself was isolated in January 2020. The rapid spread of the coronavirus around the world forced the WHO to declare a pandemic on 11 March 2020 [1].

Infection with new coronavirus is particularly dangerous for the elderly and for people with cardiovascular disease, obesity, diabetes, chronic respiratory disease, cancer, or decreased immunity [2]. The presence of such comorbidities requires the use of different drugs, and these can affect the response to viral infections in a number of ways. A good example is the use of statins, which are commonly prescribed for high-risk patients worldwide because of their beneficial effect on cardiovascular events [3].

Recent studies indicate that the use of statins lowered mortality by 42% in hospitalized patients with COVID-19 (aHR = 0.58 with (0.43–0.8) 95% CI; *p* = 0.01); they were also associated with a 20% lower risk of *acute respiratory distress syndrome* (ARDS) (IRR = 0.80 with (0.67–0.96) 95% CI; *p* = 0.016) and 49% lower risk of mechanical ventilation (aHR = 0.51 with (0.34–0.78) 95% CI; *p* = 0.02). Interestingly, statin users were significantly older (66.0 vs. 57.0 years of age, *p* < 0.001), and were more likely to demonstrate comorbidities including hypertension (81.5% vs. 30.3% *p* < 0.001), diabetes (34.0% vs. 14.6% *p* < 0.001), coronary heart disease (36.3% vs. 5.7% *p* < 0.001), cerebrovascular disease (8.8% vs. 2.3% *p* < 0.001) compared to non-users [4]. A meta-analysis by Kow et al. including 8990 COVID-19 patients found statins reduce the risk of fatal or severe disease by 30% [5]. It should be emphasized that most of the studies concerned the chronic use of statins prior to SARS-CoV-2 infection—there is still insufficient evidence of the benefits of initiating such therapy *de novo* in COVID-19.

These results may be caused by the pleiotropic activity of statins, and recent studies suggest various mechanisms that may directly affect SARS-CoV-2 endocytosis (ACE2), replication (main protease and RNA polymerase) or indirect mechanisms unrelated to coronavirus infection, such as e.g., anti-inflammatory, anti-coagulant effects or endothelial function improvement [6,7,8,9,10,11]. In addition, it is very likely that the direct effect on the ACE2 receptor could be of particular importance for the new British coronavirus strain, possibly due to the stronger interaction between the spike protein and the ACE2 receptor [12].

Despite the benefits of this therapy, statin use is associated with a number of side effects, including myopathies, elevated hepatic enzymes and increased risk of diabetes. In addition, some comorbidities of COVID-19 patients, such as nephropathy, diabetes or multiorgan failure, may also affect the efficiency and safety of this therapy [13]. However, Xiao-Jying et al. report that statin use in hospitalised COVID-19 patients was not associated with the increase of creatinine kinase (CK > upper limit of normal ULN aHR 0.97 (0.8–1.17) *p* = 0.715) or alanine transaminase (ALT > 3ULN aHR 0.98 (0.76–1.26) *p* = 0.852) [4].

The aim of this review is to collect the latest research on statin therapy in patients with COVID-19. Primarily, we aim to analyze potential mechanisms of statins favorable effect observed in patients with SARS-CoV-2. We also aim to discuss possible concerns regarding the continuation of these lipid lowering drugs during COVID-19.

## 2. Lipids in SARS-CoV-2 Infection

Being major components of cellular and viral membranes, lipids are undoubtedly involved in viral infections. Membrane/lipid rafts, i.e., cholesterol-rich subdomains of plasma membranes, are crucial elements for membrane fusion and endocytosis. An in vitro study found ApoE-mediated cholesterol influx to cause ACE2 translocation to lipid rafts, and depletion of cholesterol with methyl-beta-cyclodextrin (MβCD) reduced the ACE2 receptor localization with lipid rafts by 70% [14]. Lipids not only facilitate membrane fusion, enabling viral cell entry, but also play crucial roles in viral envelopment, replication and exit, i.e., the further steps of viral invasion [15]. Viruses are able to alter the host lipid metabolism to produce fatty acids for their own use; in previous studies, inhibition of fatty acid synthesis was associated with a significant decrease in viral replication [16]. In addition, cholesterol depletion by pretreatment of Vero E6 cells with MβCD was found to cause a significant decrease in the production of SARS-COV-1 particles by infected cells in vitro; this effect was reversed after cholesterol was added to the cellular medium, indicating that the observed reduction of virus particle release was caused by the loss of cholesterol in the cell membrane [17]. Extracting cholesterol from human embryonic kidney 293T cell membranes with MβCD reduced the entry of retroviruses pseudotyped with the SARS-CoV-2 S proteins (SARS2-PV) by 90% [14].

In addition, metabolomic studies suggest that lipids are strongly associated with the host response to SARS-COV-2 infection. Barberis et al. report that the severity of the disease was characterized by the activation of gluconeogenesis and the metabolism of porphyrins, which play a crucial role in the progress of the infection. Down-regulation of glycerophospholipids and upregulation of lysophospholipids, arachidonic and oleic acids was observed in sera of COVID-19 patients, indicating that phospholipase A2 (PLA2) is involved in COVID-19 pathogenesis and progression [18]. The activity of PLA2 stimulates the increase of inflammatory lipid mediators such as prostaglandins, leukotrienes and lysophospholipids; these may also play a crucial role in the regulation of inflammatory response, which can influence the prognosis of COVID-19 patients [19]. Moreover, the inhibition of cytosolic phospholipase A2 (cPLA2) with the use of the low-molecular-weight nonpeptidic inhibitor pyrrolidine-2 (Py-2) blocked the replication of *Coronaviridae* viruses [20].

Patients with SARS-COV 2 infection experience serum lipid disturbances. Studies have found TC, LDL-C and HDL-C levels to be significantly lower in COVID-19 patients than in uninfected patients. Lipid levels were also correlated with the COVID-19 severity. Patients with median total cholesterol 173 mg/dL (148, 204) tended to demonstrate a mild COVID course, those with 167 mg/dL (138, 197) demonstrated a severe course, while those with 125 mg/dL (95, 162) demonstrated a critical course (*p* < 0.05). Similarly, for LDL-C, median 91 mg/dL (76, 104) was associated with a mild COVID course, 86 mg/dL (69, 102) with a severe course and 69 mg/dL (48, 81) with a critical course (*p* < 0.02). For HDL-C, median 50 mg/dL (42, 59) was associated with a mild course, 50 mg/dL (41, 59) with a severe course and 36 mg/dL (29, 43) with a critical course (*p* < 0.05). For triglicerides, median 150 mg/dL (124, 213) was associated with a mild COVID course, 142 mg/dL (89, 189) with a severe course and 115 mg/dL (88, 186) with a critical course (*p* < 0.01) [21,22].

Low HDL was suggested to be an independent risk factor for a severe course of COVID-19. COVID-19 patients with low HDL at admission (median, 27 vs. 31 mg/dL, *p* = 0.032) had nearly a three-fold greater risk of developing a severe course of the disease than those with high HDL-C (HR 2.827, 95% CI 1.190–6.714, *p* = 0.019). This may be helpful in identifying patients at high risk of critical COVID-19 course who need more intense monitoring [23]. Unfortunately, the authors did not consider lipid lowering therapy in their analyses; however, these studies were performed on a Chinese population, where the use of statins is not common, even in secondary prevention [24].

Association of COVID-19 course and hypolipidemia may be supported by the fact, that SARS-CoV-2 requires lipids for the infection. There is no evidence that statins by lowering lipids may exacerbate COVID-19. On the contrary, in retrospective analysis of 170 hospitalized COVID-19 patients the use of statins prior to admission was associated with reduced risk of severe course of the disease by 70% (adjusted OR 0.29, 95%CI 0.11 to 0.71, *p* < 0.01) [25]. Statins, as hypolipidemic drugs may decrease the infectivity of SARS-CoV-2 by disrupting lipid rafts and lowering membrane cholesterol levels [26]. However statins are not only associated with lowering lipid levels, they also exert pleiotropic effects such as attenuating inflammation and atherosclerotic plaque stabilization which may be crucial in patients with atherosclerosis and COVID-19 [27]. The issue of atherosclerotic plaque stability should be more investigated in patients with SARS-CoV-2 infection.

## 3. Direct Effect of Statins on SARS-CoV-2 Endocytosis via ACE2 Protein

Endocytosis is a process in which various particles are internalized into cells. This process is also used by SARS-CoV-2 to invade human cells. One of the endocytic route for the virus is via ACE2 (angiotensin-converting enzyme-2) receptors localized in lipid rafts. Spike protein of SARS-CoV-2 attaches to ACE2 receptor which enables fusion of viral and human cellular membranes [26]. It has been previously proven that ACE2 knockout mice infected with SARS-CoV-1 had significantly lower viral replication, spike protein RNA level and lung injury in comparison to SARS-CoV-1 infected wild type mice [28]. ACE2 protein is expressed in various tissues in 2 functional forms; first-anchored in a cellular membrane which is a SARS-CoV-2 receptor and a soluble form without transmembrane domain. Both forms of ACE2 proteins take part in renin-angiotensin-aldosterone system regulation as well as in SARS-CoV-2 entry [29]

ACE2 protein is also an important part of the Renin Angiotensin Aldosterone system (RAA), which is not only associated with hypertension development, but also plays a role in balancing the inflammatory response. In human cells, ACE2 protein acts as a peptidase, converting angiotensin II to angiotensin 1–7, which exerts an anti-inflammatory, vasodilatatory and antifibrotic effect via the ACE2/Ang-(1–7)/Mas axis [30]. The failure for angiotensin II to be converted by ACE2 may lead to acute lung injury via its interaction with the AT1 receptor [28]. In previous studies with respiratory syncytial virus (RSV), ACE2 protected lungs from injury in animal models, as well as in a pediatric population [31]. Interestingly, in SARS-CoV animal models, ACE2 levels significantly decreased, supporting the idea that it may be involved in the host inflammatory response [28].

Recently, there is an ongoing clinical trial testing the use of human recombinant soluble ACE2 (hrsACE2) in severe COVID-19 patients neutralising viral S protein and reducing organ damage by counterbalancing the inflammatory response [32]. In previous in vitro studies, hrsACE2 reduced the SARS-CoV-2 viral load by a factor of 1000–5000 in cell-culture experiments [33].

In previous experimental studies, atorvastatin (5 mg/kg/day *p.o.* for three weeks) increased the occupancy of histone H3 acetylation (H3-Ac) mark on the ACE2 promoter region in heart tissue of rabbits with a high cholesterol diet (HCD—2% for 12 weeks) indicating direct or indirect epigenetic up-regulation of ACE2 by atorvastatin [34]. It is also possible that statins could up-regulate ACE2 by acting as agonists for the peroxisome proliferator γ receptor (PPAR-γ). [35].

The potential of statins as COVID-19 therapeutic agents could be supported by their ability to enhance ACE2, thus counter-balancing inflammation. The potential for statins to alleviate the hyperinflammatory response is also supported by previous experience gained from Ebola, where statins and ARBs improved patient prognosis and decreased mortality [36]. On the other hand, all steps should be taken with caution, because elevated levels of ACE2 may theoretically increase the ability of SARS-CoV-2 to infect human cells.

## 4. Statins Possibly Attenuate SARS-CoV-2 Replication Directly via Main Protease and RNA-Dependent RNA Polymerase

The main protease of SARS-CoV-2 (called M^pro^ or 3CL^pro^) is responsible for the creation of functional viral proteins by cutting the polyproteins translated from viral RNA. Many studies suggest that inhibition of M^pro^ may be a key element of COVID-19 therapy, and there is considerable interest in identifying drugs that can achieve this beneficial effect [37,38,39,40,41]. A recent in silico comparative analysis of binding energies of SARS-CoV-2 M^pro^ (6UL7) with seven statins (atorvastatin, rosuvastatin, simvastatin, pravastatin, pitavastatin, lovastatin, fluvastatin) and three common antiviral drugs (lopinavir and nelfinavir as protease inhibitors and favipiravir as inhibitor of the RNA-dependent RNA polymerase) found that the tested statins have similar binding energies than the indicated antiviral drugs, with pitavastatin being even lower. In addition, the docking results showed that pitavastatin, rosuvastatin, lovastatin and fluvastatin were the most likely to inhibit M^pro^ [9]. These results indicate that statins are likely to exert a similar or even greater inhibitory effect on the main SARS-CoV-2 protease than antiviral drugs. However, further studies are necessary to confirm this thesis.

The major polymerase responsible for SARS-CoV-2 RNA replication is RNA-dependent RNA polymerase (RdRp). Baby et al. (2020) reported that pitavastatin binds strongly to the active site of this enzyme, as demonstrated by molecular dynamics simulation [10].

The above results offer strong evidence that statins may have an additional beneficial effect on patients with COVID-19 by specifically inhibiting replication of the virus. Future in vitro and in vivo tests must be performed to confirm these in silico results.

## 5. Statins May Reduce Inflammation in COVID-19: Interleukin-6 and Toll-Like Receptor 4

COVID-19 is a severe inflammatory disease that affects the entire body, especially the respiratory system, causing severe interstitial pneumonia. According to the latest guidelines [42], treatment should depend on the phase of the disease: initial treatment is based on inhibiting viremia, and later treatment on inhibiting the strong inflammatory process called the cytokine storm.

One of the most important cytokines in COVID-19 is pro-inflammatory interleukin-6 (IL-6) [43], whose serum level strongly correlates with the severity of the disease [44]. It is known to be involved in the immune system response to many viral infections, including influenza, rabies, HBV, HCV and HIV [45]. However, the SARS-CoV-2 infection is the first to be treated with tocilizumab, an anti-IL-6 monoclonal antibody [46].

High levels of IL-6 in serum may contribute not only to a cytokine storm, but even to macrophage activation syndrome (MAS) [47]: a severe inflammation caused by activated macrophages, manifested by fever, hyperferritinemia, hypofibrinogenemia, coagulopathy and cytopenia [48]. Therefore, it is critical to ameliorate the immune response in severe COVID-19.

It has been proven that statins may reduce inflammation through their pleiotropic effect [49]. A meta-analysis of 6214 patients with heart failure from 19 randomized clinical trials showed that statins are able to lower serum levels of both IL-6 and CRP, with a prominent predominance of lipophilic statins, e.g., atorvastatin, simvastatin and pitavastatin [50].

It has been suggested that statins may lower IL-6 levels, and thus improve the prognosis of COVID-19 patients, by inhibiting Toll-like receptor 4 (TLR-4). Its stimulation may enhance the inflammatory response and the levels of pro-inflammatory cytokines, including IL-6 and TNF-alpha, via two pathways related to NF-kB: the myeloid differentiation primary response 88 (MyD88) dependent pathway and Toll/IL-1R domain-containing adaptor-inducing IFN-β (TRIF) dependent pathway. Moreover, murine models show that TLR4 inhibition acts as a protective factor against acute lung injury caused by viral infection [51], which is of particular interest in severe COVID-19 pneumonia.

Interestingly, inhibiting TLR4 could directly affect the potential for infection with the new coronavirus. An in silico study by Choudhury et al. indicates that the spike glycoprotein of SARS-CoV-2 is phylogenetically close to bat coronavirus and strongly binds with ACE2 receptor protein from both human and bat origin; the findings also suggest that cell surface TLRs, especially TLR4, are most likely to be involved in recognizing molecular patterns from SARS-CoV-2 and inducing inflammatory responses. Therefore, selective targeting of TLR4-spike protein interaction by designing competitive TLR4-antagonists could pave the new way to treating COVID-19 [52].

According to literature data, atorvastatin (0.1, 1 and 10 μM after 24 or 48 h) may reduce TLR 4 expression and inhibit the Toll-like receptor (TLR)-MYD88-NF-кB pathway in murine pro-B cell lines transfected with hTLR4/MD2 and MyD88/hTLR4/MD2 systems [11]. These outcomes suggest statin (especially atorvastatin) therapy can attenuate SARS-CoV-2 infection and prevent hyperinflammation by inhibiting TLR4.

## 6. Statins Can Possibly Attenuate Macrophage Activation Syndrome

The main problems faced by patients with severe COVID-19 are acute respiratory distress syndrome (ARDS) and systemic hyperinflammation, i.e., a cytokine storm [53]. A proper understanding of pathophysiological mechanisms is crucial.

A postmortem analysis of lung and bone marrow biopsies of 33 patients with severe COVID-19 by Prieto-Pérez et al. [54] found most bone marrow samples to demonstrate hemophagocytosis, T lymphocytosis (CD8+), and an increased percentage of myelocytes + metamyelocytes. All lung tissue samples showed diffuse alveolar damage, together with the formation of a hyaline membrane and strong infiltration by macrophages and CD8+ lymphocytes. Additionally, in many samples, thrombosis was found in the alveolar microcapillaries.

These pathological findings, together with severe systemic inflammation, suggest the occurrence of secondary hemophagocytic lymphohistiocytosis (sHLH), also known as macrophage activation syndrome (MAS). It is a life-threatening condition caused by large-scale activation of macrophages, which produce large amounts of pro-inflammatory cytokines, including IL-1, IL-6 and TNF-α. Clinically, it is often manifested by fever, hyperferritinemia, hypofibrinogenemia, coagulopathy and cytopenia [55]. Inhibiting this process may be beneficial in COVID-19 therapy.

It is known that cholesterol-lowering treatment based on statins can also reduce the migration of macrophages and their proliferation in atherosclerotic plaque [56]. Two-month rosuvastatin therapy (10–20 mg/day) may promote the differentiation of monocytes into anti-inflammatory M2 macrophages in patients with coronary disease caused by PPAR-γ activation [57].

Interestingly, Fu et al. [58] report that the stimulation of monocytes with statins such as fluvastatin, atorvastatin, rosuvastatin or simvastatin (10 µg/mL; 24 h) in the macrophage differentiation phase results in a strong subsequent inflammatory response, characterized by secretion of IL-1β and IL-6, due to LPS stimulation (100 ng/mL; 24 h); without statin stimulation, the macrophages are not able to secrete these cytokines.

In addition, statin stimulation alone had no effect on the inflammatory response of monocytes. This effect is related to the inhibition of the geranylgeranylation of the isoprenoid pathway and subsequent activation of Rac1. These findings suggest that statins may allow macrophages to remain in an activatable “monocyte-like” state, which authors call the *retainment effect*. In this state, the macrophage response may be more suited to the provoking factors, and thus more immunocompetent.

Although statins may affect the inflammatory properties of macrophages, there is still no evidence that they have a beneficial effect on MAS. Further analysis of the effects of statins on COVID-19 and MAS patients is needed.

## 7. Statins Improve Endothelial Functions

The vascular endothelium not only acts as the first barrier between blood and other tissues, but also as a regulator of hemostasis through the synthesis and secretion of procoagulant and anticoagulant factors. Recent studies show that SARS-CoV-2 can affect the endothelium, induce inflammation and provoke thrombosis [59,60,61]. Post mortem examination of COVID-19 patients revealed increased expression of activated complement components. Both systemic complement activation and ACE2 reduction may lead to microvasculopathy and severe thrombosis, which may be an important element of disease progression [62]. Since statins can protect the endothelium, they can potentially suppress the harmful effect of coronavirus. Clearly, these drugs have a range of beneficial effects on the vascular endothelium, independent of their hypolipidemic effect [6].

The pleiotropic effect of statins is based mainly on the reduction of reactive oxygen species (ROS): a harmful factor leading to endothelial dysfunction and the development of atherosclerosis [63,64]. ROS inhibition is achieved by the reduction of miRNA-221 and miRNA-222 and the promotion of the BH4 cofactor, which is associated with the uncoupled form of endothelial nitric oxide synthase 3 (eNOS) [65,66]. Statin treatment can also counteract the effect of disturbed blood flow, which raises ROS levels by increasing high shear stress in the endothelium; the treatment stimulates Krüppel-like Factor 2 (KLF-2) and activates cystathionine γ-lyase (CSE) [67,68].

In addition, statins can attenuate NOD-, LRR- and pyrin domain-containing protein 3 (NLRP3) Inflammasome, a potent pro-inflammatory structure in endothelial cells, by stimulating pregnane X receptor (PXR) [69].

When the endothelium is clearly dysfunctional as a result of severe damage, the regeneration process becomes crucial. The process is supported by human endothelial progenitor cells (EPCs), as they naturally replace damaged cells. EPCs have also been found to play an important role in the prevention and treatment of cardiovascular disease [70]. It has been reported that statins can increase EPC levels after ischemic heart failure, a further beneficial pleiotropic element of their use [71].

## 8. Statins May Reduce the Risk of Thrombosis in COVID-19

A multicentre retrospective study found the overall thrombotic complication rate associated with SARS-CoV-2 infection to be 9.5% (95% CI 6.8–12.8) [72]. One of the most serious thrombotic complications is pulmonary embolism (PE), which is also more common in COVID-19 patients [73,74]. Since case studies suggest that PE may occur even after COVID-19 patients are discharged from hospital [75], prevention of thrombosis is an important aspect of treatment. Statins may reduce the risk of PE during SARS-CoV-2 infection due to their anticoagulant effects [76].

A prospective multicentre cohort study [77] showed that statin users have a lower risk of recurrent venous thromboembolism (rVTE) than non-users; however, this effect was only seen during anticoagulant-free periods. The adjusted hazard ratio (aSHR) was 0.50 (95% CI 0.27 to 0.92, *p* = 0.03); however, this effect was enhanced by the use of propensity scores (aSHR 0.20, 95% CI 0.08 to 0 49, *p* < 0.001).

Another cohort study [78] involving 3093 patients (median follow-up: 1529 days) found statin treatment to be associated with a lower risk of recurrence of pulmonary embolism (aSHR 0.50, 95% CI: 0.36–0, 70) with or without VKA (vitamin K antagonist) anticoagulation. In addition, the analysis showed that the strongest effects were seen with the most potent statins, i.e., atorvastatin and rosuvastatin.

A potential explanation for this phenomenon is the effect of statins on plasminogen activator inhibitor-1 (PAI-1). A meta-analysis of 16 randomized controlled trials showed that statins (especially atorvastatin) can significantly reduce the PAI-1 serum level [79], thus increasing the degradation of fibrin clots by the enzyme plasmin.

## 9. Statins May Alleviate Post-COVID Pulmonary Fibrosis

There is growing evidence that pulmonary fibrosis can occur after ARDS (Acute Respiratory Distress Syndrome) in some patients suffering from COVID-19. It is estimated that post-COVID fibrosis may affect even one third of hospitalised patients [80]. Fibroproliferation occurs in some patients after the inflammatory phase of ARDS as a result of tissue damage and failure to repair it [81]. The CT image of fibrosis correlates with the quality of life and respiratory functions of patients after recovery from ARDS [82]. However not all patients recovering from ARDS develop pulmonary fibrosis.

Statins may have a beneficial effect on various factors that promote lung fibrosis following ARDS, such as endothelial dysfunction, VEGF, IL-6 and TNFα [80]. They have been found to improve endothelial function, exert an anti-inflammatory effect and lower the expression of VEGF [83]. Simvastatin improved 28-day survival in patients with hyperinflammatory ARDS [84]. It is also believed to suppress epithelial-mesenchymal transition (EMT) by attenuating TGF-β signalling, known to be associated with post infectious pulmonary fibrosis by causing remodelling and connective tissue deposition among fibroblasts and epithelial cells [85,86]. It has also been found to cause fibroblast apoptosis [87]. Pravastatin was also found to exert a beneficial effect on a model of bleomycin-induced lung injury and fibrosis by influencing other factors involved in lung fibrosis, including connective tissue growth factor, RhoA and cyclin D1 [88].

Statins have also been reported to have beneficial effect in IPF (Idiopathic Pulmonary Fibrosis). Statin users with IPF had lower mortality and lower risk of hospitalization than non-users with IPF [89].

## 10. The Safety of Statins

Despite the many benefits of statin treatment, such as reducing mortality and cardiovascular events, statin treatment has been found to have side effects.

A common problem among statin users is related to muscle tissue [90]. Their use can cause myalgia (5% of patients), a muscle pain that hinders everyday functioning. Less frequent are myopathies (5/100,000 patients-year) or muscles injuries, characterized by an increase in creatine kinase (CK) levels and requiring treatment modification by dose reduction or drug change. The most dangerous, and the rarest, complication is rhabdomyolysis (0.1–8.4/100,000 patients-year): a potentially fatal condition associated with extensive damage to many muscles and renal dysfunction.

Such conditions are not unknown in COVID patients. Studies report that 34.8% of 138 COVID-19 hospitalized patients from Wuhan (China) had myalgia [91], and 2.8% of 615 COVID-19 patients from Milan (Italy) had myalgia or arthralgia; however, 28.6% demonstrated elevated serum CK levels [92]. In contrast, a study revealed no differences in serum CK levels between 1219 patients taking statins and 12,762 not taking statins [4]. The presence of elevated CK levels may increase the risk of statin myopathies, including rhabdomyolysis; for example, one study reported a case of fatal rhabdomyolysis in a 57-year-old woman suffering from COVID-19 with hypertension, obesity (class II) and dyslipidemia, who was taking rosuvastatin (5 mg/day) [93].

The risk of myopathies may be increased by drug interactions between statins and certain anti-infective drugs (e.g., itraconazole, erythromycin), calcium antagonists (verapamil, diltiazem, amlodipine) or others (amiodarone, cyclosporine) [3]. In addition, drugs used to treat COVID-19 may also require statin therapy to be modified. According to a statement by HEART UK experts, atorvastatin cannot be combined with remdesivir and should be changed to rosuvastatin [94]. Tocilizumab must not be combined with any statin, in such case statin therapy should be temporarily suspended. Dexamethasone is not a contraindication to statin therapy. Therefore, any decision to administer a statin must be considered on an individual basis, considering the risks and benefits for each patient.

It is highly important to monitor patients with SARS-CoV-2 infection who are taking statins to prevent serious muscular side effects. Further analysis is needed to assess the scale of the problem.

Another common side effect of statins is disturbed carbohydrate metabolism and an increased risk of diabetes (odds ratio 1.09; 95% CI 1.02–1.17). A meta-analysis of 91,140 patients found that 255 must take a statin for at least four years to cause one additional episode of diabetes [95]. This effect may be stronger in SARS-CoV-2 patients, as systemic COVID-19 inflammation is associated with deterioration of glycaemia control [96]. Potent glucocorticoid therapy is also a risk factor for glycaemic disorders.

Interestingly, the CORONADO study found routine statin treatment before hospitalization was significantly associated with increased seven-day (12.8% vs. 9.8%, respectively; *p* = 0.02) and 28-day (23.9% vs. 18.2%, respectively; *p* < 0.001) mortality in 2449 type 2 diabetes mellitus (T2DM) patients hospitalized for COVID-19 [13]. Inverse probability of treatment weighting (IPTW) emphasized the association between statin therapy and deaths within seven days (OR (95% CI): 1.74 (1.13–2.65)) and 28 days (OR (95% CI): 1.46 (1.08–1.95)).

However, other studies do not support these results. A study of 4252 COVID-19 patients, including 2266 with type 2 diabetes, found statin treatment to be associated with lower serum CRP levels (10.2; interquartile range (4.5–18.4) versus 12.9; interquartile range (5.9–21.4) mg/dL; *p* < 0.01) and reduced cumulative in-hospital mortality (24% versus 39%; *p* < 0.01) [97]. Interestingly, this effect was not observed for non-diabetes patients.

Statins may also increase the levels of the liver enzymes aspartate aminotransferase (AST) and alanine aminotransferase (ALT), but these elevations are often clinically insignificant [3].

Unfortunately, 58% of COVID-19 patients demonstrate elevated levels, and these can be as much as two times above the upper limit [98]. However, some studies suggest that statin use has no significant effect on serum ALT levels in COVID-19 patients [4]. It is still unclear whether this elevation is a direct effect of the virus or an indirect systemic inflammatory effect, and indeed whether the increase is clinically significant. Nevertheless, liver function should be monitored in patients, especially those using remdesivir, which causes a strong increase in transaminase and bilirubin levels, and can lead to hepatocellular injury.

Despite evidence that lower cholesterol concentrations are associated with more severe course of COVID-19, there is, however, no evidence that statins may worsen prognosis. On the contrary, these drugs may reduce the pro-inflammatory and pro-thrombotic mechanisms that characterize more severe cases of COVID-19 [27]. Currently, there is no evidence to support discontinuation of statins in patients with COVID-19, except when important elevations of hepatic enzymes, rhabdomyolysis, or drug-attributed risk of life occur. On the other hand, there is no indication for the use of these drugs specifically to prevent complications of SARS-CoV-2 infection.

Moreover, HEART UK experts emphasize that the benefits of this therapy outweigh the risks and should be continued in patients at high cardiovascular risk who develop COVID-19 with closely monitored liver enzyme and drug interactions [94].

## 11. Summary

The COVID-19 pandemic has driven an ongoing search for easily-available yet effective therapeutic agents. The benefits of statins, and their positive effects on the mortality and prognosis of COVID-19 patients, has prompted consideration of their possible mechanisms. Statins can affect SARS-CoV-2 directly by inhibition of the viral replication by decreasing the activity of main protease (Mpro), and RNA-dependant RNA polymerase (RdRp). The indirect benefits of statin use in SARS-CoV-2 infection may be associated with their immunomodulatory effects, such as counter-balancing the inflammatory response through ACE2, lowering the levels of inflammatory markers such as Il-6 through TLR-4 inhibition, and improving endothelial function. Statins may also improve the immunocompetence of the macrophage response, which could be especially favourable in Macrophage Activation Syndrome. Moreover, statin treatment may also be beneficial to COVID patients by reducing the risk of complications, such as thrombosis, by reducing the serum level of PAI-1, and pulmonary fibrosis, by attenuating TGF-β signalling. Although statins are considered rather safe for use, some adverse effects may occur, such as myopathy, carbohydrate metabolism deterioration and liver enzyme elevation.

The effectiveness of statins in COVID-19 patients with high cardiovascular risk indicates that they should not be withdrawn during the infection. Currently, there is no evidence from randomized controlled trials which would clearly support statins *de novo* initiation in SARS-CoV-2 infection. However the results of an ongoing clinical trial with Atorwastatin 40 mg as an adjunctive therapy of COVID-19 (STATCO19, NCT04380402) will dispel some doubts on this issue.

## Data Availability

Not applicable.

## Abbreviations

SARS-CoV-2	Severe Acute Respiratory Syndrome Coronavirus 2
COVID-19	Coronavirus Disease 2019
WHO	World Health Organisation
aHR	adjusted Hazard Ratio
CI	Confidence Interval
ARDS	Acute Respiratory Distress Syndrome
IRR	Incidence rate ratio
LDL	Low Density Lipoprotein
ACE2	Angiotensin Converting Enzyme 2
TLR	Toll-like receptor
CK	Creatine Kinase
ALT	Alanine Transaminase
ULN	Upper Limit Norm
MβCD	Methyl-beta-cyclodextrin
ApoE	Apolipoprotein E
PLA2	Phospholipase A2
SARS-PV	retroviruses pseudotyped with SARS-CoV-2 S proteins
cPLA2	cytosolic phospholipase A2
Py-2	pyrrolidyne 2
TC	total cholesterol
HDL	High Density Lipoprotein
RNA	ribonucleic acidSARS-CoV-1—Severe Acute Respiratory Syndrome Coronavirus 1
RAA	Renin Angiotensin Aldosterone
AT1R	Angiotensine 1 receptor
RSV	Respiratory Syncytial Virus
H3-Ac	histone H3 acetylation
HCD	High Cholesterol Diet
PPAR-γ	peroxisome proliferator γ receptor
ARBs	Angiotensin Receptor Blockers
Mpro	main protease
RdRp	RNA-dependent RNA polymerase
HBV	Hepatitis B Virus
HCV	Hepatitis C virus
HIV	Human Immunodeficiency Virus
IL-6	interleukin 6
MAS	Macrophage activating syndrome
CRP	C reactive protein
MyD88	Myeloid differentiation primary response 88
TRIF	Toll/IL-1R domain-contactiong adaptor-inducing IFN-βTRIF—Toll/IL-1R domain-contactiong adaptor-inducing IFN-β
IFN-β	Interferon beta
NF- κB	nuclear factor kappa-light-chain-enhancer of activated B cells
CD8+	cluster of differentiation 8
sHLH	secondary hemophagocytic lymphohistiocytosis
TNF-α	Tumor Necrosis Factor alpha
LPS	Lipopolysaccharides
ROS	reactive oxygen spieces
miRNA-221	MicroRNA-221
eNOS	endothelial nitric oxide synthase
KLF-2	Krüppel-like Factor 2
CSE	cystathionine γ-lyase
NLRP3	NOD-, LRR- and pyrin domain-containing protein 3
PXR	pregnane X receptor
EPCs	Endothelial Progenitor Cells
PE	pulmonary embolismr
VTE	recurrence of venous thromboembolism
aSHR	adjusted Hazard Ratio
VKA	vitamin K antagonist
PAI-1	plasminogen activator inhibitor –1
CT	computed tomography
VEGF	Vascular endothelial growth factor
TGFβ	transforming growth factor β
IPF	Idiopathic Pulmonary Fibrosis
OR	Odds Ratio
AST	aspartate aminotransferase
